# Prevalence and predictors of work-related musculoskeletal disorders among workers of a gold mine in south Kivu, Democratic Republic of Congo

**DOI:** 10.1186/s12891-020-03828-8

**Published:** 2020-12-01

**Authors:** Alfred Okello, Solomon Tsebeni Wafula, Deogratias K. Sekimpi, Richard K. Mugambe

**Affiliations:** 1 Department of Public Health, St Mary’s Hospital Lacor, Gulu, Uganda; 2 Department of Disease Control and Environmental Health, Makerere University School of Public Health, Kampala, Uganda

**Keywords:** Work-related musculoskeletal disorders, Ergonomics, Mining workers, DR Congo

## Abstract

**Background:**

Work-related musculoskeletal disorders (WRMSDs) are a major constraint to worker performance and health. However, research on their prevalence and associated factors among workers at gold mines in the Democratic Republic of Congo (DRC) is insufficient. The present study aimed to determine the prevalence and predictors of WRMSDs among workers of a Gold Mine in South Kivu, DRC.

**Methods:**

Cross sectional data on prevalence of WRMSDs and risk factors was collected using a modified Nordic questionnaire and upper limb Core QX checklist from 196 workers of a gold mine. WRMSDs were defined as pain or injury(ies) or discomfort, numbness or limitation of movement in the musculoskeletal system at any time in the past 12 months which lasted at least 24 h. These had to be either induced or aggravated by work and circumstances of its performance. A generalised linear model of the Poison family with link log and robust error variances was used to generate prevalence ratios (PRs) and 95% confidence intervals (CIs) for the factors associated with WRMSDs. The effect of individual, ergonomic and psychosocial factors on WRMSDs were investigated while controlling for known confounders.

**Results:**

Most workers were males 187 (95.4%) and their age ranged between 23 and 60 years with mean of 36.3 years. Of the 196 workers, 49 (25.0%) reported having at least one WRMSD during the previous 12 months. WRMSDs with highest occurrence rate were the lower back pain (14.8%), followed by thighs/hip pain (9.2%) and shoulder pain (8.2%). Prolonged heavy lifting/shovelling shovelling (*PR* = 1.69, 95% CI [1.32, 3.24] and longer work shifts (> 9 h) (*PR* = 3.56, 95% CI [1.76, 16.58]) were predictive for WRMSDs while jobs with low demands were protective against WRMSDs (*PR* = 0.18, 95% CI [0.08, 0.44]).

**Conclusion:**

The prevalence of WRMSDs is high and associated with prolonged heavy lifting/shovelling, longer work shifts and job demands. We recommend lowering workload and job demands and improving work ergonomics to mitigate and prevent the WRMSDs among workers in goldmines.

## Background

Musculoskeletal disorders (MSDs) have over the years become major health conditions worldwide resulting in increased burden on general medical care. MSDs affect all parts of the body with the back, neck and shoulders and upper limbs accounting for more than 50% of cases [1–3]. They have negative impacts on quality of life such as work-related disability as well as substantial financial implications related to medical expenses and workers’ compensation [4]. In gold mining, the working conditions are physically demanding and dangerous due to heavy and awkward loads, unstable underground structures, heavy tools and equipment, among other factors often leading to MSDs [5–7].

Work-related musculoskeletal disorders (WRMSDs) resulting from occupational activities present a bigger problem than usually estimated [8, 9]. WRMSDs are the most expensive occupational conditions and they are the leading work-related health concern, in high income as well as low and middle income countries, accounting for over 30% of all injuries and requiring time away from work [10]. WRMSDs are highly prevalent in many African countries with the prevalence of any MSD ranging from 15 to 93.6% [11, 12]. About 20% of the overall prevalence is contributed by the industrial sector which includes mining [12]. Studies conducted amongst workers of gold mines in South Africa and Ghana found the prevalence of MSDs to be 65.3 and 85% respectively [7, 10]. The prevalence of MSDs usually varies between studies due to lack of standard/uniform definitions of MSDs, leading to variations in case definitions and data collecting procedures across studies. The differences in studied populations e.g., by economic activity, further demonstrates the disparities [1, 2, 12, 13]. The Occupational Safety and Health Administration has estimated that WRMSDs are understated by at least a factor of two based on studies and experience [10] .

The etiology and pathogenesis of WRMSDs are complicated and multifactorial. Several factors have been extensively investigated in different occupations [8]. The risk factors of MSDs can be biomechanical, psychosocial, or individual [14]. These risk factors vary over time and in different occupational situations and usually interact with each other to create an elevated risk [7, 8, 15]. The mining workplace may have risk factors related to equipment/vehicle design; work organization (consisting of high job demands; time pressures; lack of job rotation and long working hours without opportunity for rest and recovery); limited access (usually in form of limited working space); duration of task; overtime; and maintenance/breakdowns of equipment [2, 7, 10].

Despite the startling global figures on WRMSDs, only a few research studies have been conducted in sub-Saharan Africa, much less in Democratic Republic of Congo (DRC) where mining, a hazardous activity, provides many of its citizens with jobs. This study, therefore, aimed to determine the prevalence of and predictors of WRMSDs amongst workers of Twangiza Mining site in south Kivu, DR Congo.

## Methods

### Study design

This was a cross-sectional study, conducted among workers in Twangiza Gold Mine, a subsidiary of Banro Corporation in the DR Congo in 2018. In 2011, Twangiza became the first commercial gold mine built in the DRC in over 50 years. It is an open pit gold mine which started commercial gold production in late 2012. It is located 35 km west of the Burundi border and 45 km to the southeast of Bukavu in South Kivu.

### Study population and sample size

The study was conducted amongst workers of Twangiza gold mine for 4 weeks from 1st June 2018 to 30th June 2018. Eligibility for participants is shown in Table 1.

**Table 1:** Eligibility criteria for recruiting participants in the Musculoskeletal disorders study in Twangiza Gold Mine

Inclusion criteria	Exclusion criteria
• Age of 18 and above • Minimum of one year experience at the mining site. • No history of trauma or injuries and psychological problems (information obtained from annual medical reports at the mine’s medical office) • Signed (or thumb-printed) informed consent document	• Child miners below 18 years of age. • Previous trauma or ongoing psychological problem (information obtained from annual medical reports at the mine’s medical office) • Refusal to consent to participate.

The gold mine employed 691 workers at the time. Workers were stratified into six departments (strata) with each performing different tasks. The different strata were defined by grouping jobs together based on similar job demands. These departments include the mining department (133 members) which does extraction and blasting of gold ore requiring high efforts, engineering (82 members) and maintenance departments (137 members) which are involved in construction and maintenance of equipment respectively thus demanding high impact efforts. Mineral resources (MRM) department (86 members) explores the ore reserves including drilling requiring high efforts. Metallurgy department (116 members) refines and processes gold ore into pure gold with moderate effort requirements. The “others” (137 members) are involved in the transportation of goods/personnel to and from the mining sites, planning, educating and maintaining health and safety for the company with minimal effort requirements.

The Kish Leslie formula (1965) was used to determine the sample size [16]. We assumed an alpha of 0.05, power (1-beta) of 0.80, a sampling error of 5%, and prevalence (P) of WRMSDs of 42.6% was considered from a similar study in Malawi [2]. The sample size was 376 but since the proportion of sample to population was larger than 5%, we then used a finite population correction formula by Daniel [17] with an additional 10% to account for refusal to participate/non-response giving an adjusted sample size of 273 [18, 19].

To make the sample size, we set out to randomly select 46 participants from each of the 6 departments (strata). Random sampling was applied instead of proportionate sampling since the departments had similar number of workers. We used a table of random numbers to select the participants per department. Complete data were obtained from 196 gold mining workers hence a response rate of 71.8%. The reasons for non-response include incomplete data, difficulties to schedule appointments since some workers had left for their regular day offs/leave while others had changed their minds not to participate.

### Data collection and measurements

A self-administered questionnaire was developed from existing surveys of musculoskeletal disorders and risk factors. Various questionnaires guided the design of the questionnaire for this study including the standardized Nordic, and the modified versions of the Washington state risk factor checklist and the upper limb Core QX checklist used by Kunda et al., [2, 20]. The Nordic questionnaire is a widely accepted, easy to administer, and cost efficient tool for collecting data on self-reported musculoskeletal discomfort and sickness absence and it has been shown to have high validity for capturing MSDs in various settings [20] and for different body regions [21, 22].

The questionnaire had four sections with the first section providing data on background variables such as age, sex, educational level. The second part consisted of questions on the MSD injury/complaints profile.

The modified version of the Nordic questionnaire measured the subjective ache/pain/numbness/injury on the different body parts. Work related MSD was defined as developing an ache/pain/numbness/injury after starting work at mines or either aggravated by working conditions while on duty (work related) during the last 12 months. A “yes” response to complaints from duty was used to ascertain the prevalence of WRMSDs. The affected body parts (Neck, shoulder, elbows, wrists/hands, upper back, lower back, hips/thighs, knee, ankles/feet) were also recorded under this section.

Section three consisted of the risk factors such as the work-environment characteristics and work-practices such as machines used, postures adopted at work and total work duration per day. The workers reported on health hazards at their job types to provide estimates of safety hazards about risk factors. The workers indicated the length of exposure to an activity which determined the exposure level as being lower risk /cautionary (occasionally/less than 2 h per day or less than 10 times per day) or higher risk / hazardous (≥2 h/day or ≥ 10 times/day).

The fourth section recorded the psychological and psychosocial risk factors which were measured using a modified version of the upper limb Core QX checklist [2]. Five questions were asked on job demand and they had responses with the format 1 = *strongly disagree*; 2 = *disagree*; 3 = *agree*; and 4 = *strongly agree*), These responses were collapsed into two categories (agree or disagree) during analysis. Agreement that some aspects of the job are demanding was considered “high job demand” or otherwise low job demand. Job security was considered present if the participant felt he/she is indispensable and less likely to lose their job, otherwise considered job insecure.

We also asked four questions on job control (variety amount, pace and duration of tasks) with responses “very little”, “little”, moderate”, “much” and “very much”. These were each collapsed into “little” and “much”. A participant was considered to have job control if they indicated much control on any of the four aspects, otherwise considered to have low job control.

Regarding work manship, four questions were asked about receiving support from supervisors with options “Very much”, much or (easy)”, “little” or “Not at all”. These were collapsed to two categories; “little” or “much”. Workmanship was then categorised as “good” if workers received much support from supervisors on any of the four aspects or bad if otherwise.

Mental state was evaluated using five questions on anxiety and depression with options rarely or none (*Coded 1*), sometimes (*coded 2*), Often (*coded3*), Most or all of the time (*coded4*). These were collapsed to two categorises (rarely/ none/sometimes and Often/most or all of the time). Mental state was considered “normal” if the participants indicated none/rarely or sometimes on any of the questions and otherwise considered not normal. The full English questionnaire for the study is provided as supplementary file 1.

Four individuals were trained as research assistants for 3 days on study aims, procedures, ethics, MSDs, associated factors of MSDs and preventive measures. They distributed and collected the questionnaires and assisted the workers with difficulties encountered during the filling of questionnaires. The research assistants were introduced to the participants and an appointment was made with the mine sectional supervisors and all the participants who were available on the day and time of questionnaire distribution. The questionnaires were distributed by the researcher or research assistants and by the sectional supervisors who were on duty. Those on the night shift had the questionnaires distributed to them by the sectional heads operating at night who had received a briefing from the daytime sectional heads.

The questionnaires were completed over a 4 weeks period with the researcher and research assistants collecting the completed questionnaires daily and also reminding those who had not yet completed to do so if possible. The original questionnaire was designed in English and later translated into French since most of the workers spoke French and the minority English.

### Statistical analysis

All generated data were entered into a Microsoft Excel database, cleaned and exported to Stata version 14.0 (StataCorp, Texas). Continuous data were expressed as mean and standard deviation. Categorical data variables such as sex, age groups, the prevalence of WRMSDs, prevalence of WRMSDs by age categories, working (shift) hours, body parts affected, department, exposure to the ergonomic and psychosocial factors were expressed as frequencies and proportions. Prevalence ratios (PRs) were computed using a multivariable modified Poisson regression with the logarithm as the link function, with robust error variances to measure the association between the WRMSDs and independent variables. Simple models consisting of the outcome and one independent variable were run to obtain the crude PRs. Variables that had *p* values under liberal threshold of 0.1 in bivariate models were included in the multivariable model [23]. Backward stepwise elimination method was applied until only variables with p value ≤0.05 and those significantly improved the fit of the model were retained. The goodness of fit test showed an insignificant *p*-value of 0.7553 suggesting that the model fitted the data reasonably well. The adjusted PRs and their 95% confidence intervals are presented.

## Results

### Demographic characteristics

The respondents were aged between 23 and 60 years old (mean = 36.3; SD =7.9 years). The work experience of the respondents ranged from 1 to 11 years (mean = 4.1; SD =2.0 years) with 72.5% (142) of the respondents having an experience of 1–5 years. A work shift ranged from 7 to 15 h (mean = 11.2; SD = 1.41). Majority 83.2% (163) of the respondents had work shifts of more than 9 h (Table 2).

**Table 2:** Demographic characteristics of participants (*N* = 196)

Characteristic	Category	*n*	Summary measure
Age in years	< 30	37	18.9%
	30–34	54	27.6%
	35–39	48	24.5%
	≥ 40	57	29.1%
	Mean (SD)		36.3 (7.9)
Sex	Males	187	95.4%
	Females	9	4.6%
Department	Engineering	32	16.3%
	Maintenance	34	17.4%
	Metallurgy	34	17.4%
	Mining	30	15.3%
	Mineral Resources Management	33	16.8%
	Others	33	16.8%
Education level	Primary	17	8.8%
	Secondary	83	42.8%
	Tertiary	94	48.5%
Working experience (years)	1–5	142	72.4%
	> 5	54	27.6%
	Mean (SD)		4.1 (2.0)
Work shifts in hours	≤ 9 h	33	16.8%
	> 9 h	163	83.2%
	Mean (SD)		11.2 (1.4)
Reported MSDs occurring in last 12 months	Reported MSD symptom	120	61.2%
	Did not report any MSD	76	38.8%
	Reported as work-related MSD	49	25.0%

*MSD* musculoskeletal disorder

### Prevalence of MSDs and WRMSDs

Of the respondents, 61.2% (120) reported having had a complaint (pain or discomfort) in some part (s) of their body within 12 months prior to the study. A quarter of the respondents 25.0% (49) reported that the complaints were work related. Table 3 show**s** that the 12-months prevalence rates of WRMSDs was highest in the lower back (LBP) 14.8%, followed by hips/thighs 9.2% and then shoulder 8.2% (Table 3).

**Table 3:** The reported body parts affected by musculoskeletal disorders

Reported complaint^a^	All MSD complaints, *n (%)*	Work-related complaints, *n (%)*
Lower back	79 (40.3)	29 (14.8)
Hips/thighs	43 (21.9)	18 (9.2)
Shoulder	23 (11.7)	16 (8.2)
Wrists/Hands	25 (12.7)	14 (7.1)
Upper back	33 (16.8)	12 (6.1)
Feet	37 (18.9)	12 (6.1)
Knees	16 (8.2)	9 (4.6)
Neck	18 (9.2)	8 (4.1)

*MSD* musculoskeletal disorder, *LBP* low back pain

^a^Multiple responses

### Predictors of work-related musculoskeletal disorders

In multivariable regression, after controlling for age of workers, no significant association was observed between individual factors and reporting WRMSDs (Table 4).

**Table 4:** Multivariable analysis of the individual predictors of work-related musculoskeletal disorders

Individual factors	Self-reported WRMSDs in the past 12 months (%)		Unadjusted Model		Adjusted model	
	Yes (*n = 49*)	No (*n = 147*)	*PR*	[95% CI]	*PR*	[95% CI]
**Age in years**						
< 30	9 (24.3)	28 (75.7)	1		1	
30–34	12 (22.2)	42 (77. 8)	0.91	[0.43, 1.95]	0.95	[0.47, 1.91]
35–39	8 (16.7)	40 (83.3)	0.69	[0.29, 1.61]	0.75	[0.33, 1.68]
≥ 40	20 (35.1)	37 (64.9)	1.44	[0.74, 2.82]	1.63	[0.86, 3.09]
**Department**						
Mining	13 (43.3)	17 (56.7)	1			
Engineering	12 (37.5)	20 (62.5)	0.87	[0.47, 1.59]		
Maintenance	3 (8.8)	31 (91.2)	0.20	[0.06, 0.65]**		
Metallurgy	8 (26.7)	22 (73.3)	0.54	[0.26, 1.13]		
Mineral Resources	12 (36.4)	21 (63.6)	0.84	[0.46, 1.55]		
Others	1 (3.0)	32 (97.0)	0.70	[0.01, 0.51]**		
**Level of education**						
Primary	5 (29.4)	12 (70.6)	1			
Secondary	18 (21.7)	65 (78.3)	0.74	[0.32, 1.72]		
Tertiary	24 (25.5)	70 (74.5)	0.87	[0.38, 1.96]		
**Sex**						
Male	48 (25.7)	139 (74.3)	1			
Female	1 (11.1)	8 (88. 9)	0.43	[0.07, 2.80]		
**Experiencein mining**						
1–5 years	29 (20.4)	113 (79.6)	1		1	
> 5 years	20 (37.0)	34 (63.0)	1.81	[1.13, 2.92]*	1.39	[0.88, 2.18]

***p < 0.001, ** *p* < 0.01, * *p* < 0.05. significant association between explanatory variables and reporting WRMSDs

*CI* confidence interval, *PR* prevalence ratios, *WRMSD* work related musculoskeletal disorders

Regarding ergonomic risk factors, workers with a work shift lasting more than 9 h were 3.56 times more likely to report a WRMSD complaint than those working less than 9 h a day (*PR* = 3.56, 95% CI [1.76, 16.58]). The ergonomic factor significantly associated with reporting a WRMSD was heavy lifting and/or lowering objects. Workers involved in heavy lifting and/or lowering/shovelling for ≥10 times /2 h per day were 1. 69 times more likely to report a WRMSD than those not involved in lifting/lowering/shovelling (*PR* = 1.69, 95% CI [1.32, 3.24]) (Table 5). Psychosocially, we found that low demanding jobs were protective with the workers being 82% less likely to report a WRMSD complaint than those with high job demands (*PR* = 0.18, 95% CI [0.08, 0.44]) (Table 6).

**Table 5:** Multivariable analysis of the ergonomic predictors of work-related musculoskeletal disorders

Ergonomic factors	Self-reported WRMSDs in the past 12 months (%)		Unadjusted Model		Adjusted model	
	Yes (*n = 49*)	No (*n = 147*)	*PR*	[95% CI]	*PR*	[95% CI]
**Age in years**						
< 30	9 (24.3)	28 (75.7)	1			1
30–34	12 (22.2)	42 (77. 8)	0.91	[0.43, 1.95]		0.94 (0.39, 2.25)
35–39	8 (16.7)	40 (83.3)	0.69	[0.29, 1.61]		0.78 (0.28, 2.18)
≥ 40	20 (35.1)	37 (64.9)	1.44	[0.74, 2.82]		2.04 (0.96, 4.37)
**Level of education**						
Primary	5 (29.4)	12 (70.6)	1			1
Secondary	18 (21.7)	65 (78.3)	0.74	[0.32, 1.72]		0.39 (0.18, 0.85) *
Tertiary	24 (25.5)	70 (74.5)	0.87	[0.38, 1.96]		0.81 (0.42, 1.57)
Experience						
1–5 years	29 (20.4)	113 (79.6)	1			1
> 5 years	20 (37.0)	34 (63.0)	1.81	[1.13, 2.92]*		1.23 (0.73, 2.07)
**Duration of the Shifts**						
≤ 9 h	2 (6.1)	31 (93.9)	1		1	
> 9 h	47 (28.8)	116 (71.2)	4.76	[1.21, 18.69] *	3.56	[1.76, 16.58] *
**Heavy/frequent lifting / lowering / shoveling**						
No exposure	17 (24.6)	52 (75.4)	1		1	
Caution	15 (16.3)	77 (83.7)	0.66	[0.36, 1.23]	0.50	[0.27, 1.04]
Hazard	17 (48.6)	18 (51.4)	1.97	[1.15, 3.37] **	1.69	[1.32, 3.24] *
**Awkward postures**						
No exposure	9 (18.8)	39 (81.28)	1		1	
Caution	15 (19.2)	63 (80.8)	1.03	[0.49, 2.16]	1.38	[0.67, 2.86]
Hazard	25 (35.7)	45 (64.3)	1.90	[0.98, 3.72]	1.26	[0.63, 2.54]
**High hand force**						
No exposure	15 (25.9)	43 (74.1)	1			
Caution	11 (17.5)	52 (82.5)	0.68	[0.34, 1.35]		
Hazard	23 (30. 7)	52 (69.3)	1.19	[0.68, 2.06]		
**Highly repetitive work**						
No exposure	18 (20.9)	68 (79.1)	1			
Caution	18 (27.3)	48 (72.7)	1.30	[0.74, 2.31]		
Hazard	13 (29.6)	31 (70.4)	1.41	[0.76, 2.61]		
**Vibrating tools**						
No exposure	23 (20.5)	89 (79.5)	1			
Caution	12 (24.0)	38 (76.0)	1.17	[0.63, 2.16]		
Hazard	14 (41.2)	20 (58.8)	2.01	[1.16, 3.45] **		
**Bouncing**						
No exposure	21 (19.6)	86 (80.4)	1			
Caution	9 (24.3)	28 (75.7)	1.24	[0.62, 2.46]		
Hazard	19 (36.5))	33 (63.5)	1.86	[1.10, 3.15] *		
**Static postures**						
No exposure	10 (16.1)	52 (83.9)	1			
Caution	22 (31.0)	49 (69.0)	1.92	[0.99, 3.74]		
Hazard	17 (27.0)	46 (73.0)	1.67	[0.83, 3.37]		
**Pushing and pulling**						
No exposure	21 (22.3)	73 (77.7)	1			
Caution	20 (28.2)	51 (71.8)	1.26	[0.74, 2.14]		
Hazard	8 (25.8)	23 (74.2)	1.16	[0.57, 2.34]

**Table 6:** Multivariable analysis of the psychosocial predictors of work-related musculoskeletal disorders

Psychosocial factors	Self-reported WRMSDs in the past 12 months (%)		Unadjusted Model		Adjusted model	
	Yes (*n = 49*)	No (*n = 147*)	*PR*	[95% CI]	*PR*	[95% CI]
**Age in years**						
< 30	9 (24.3)	28 (75.7)	1			1
30–34	12 (22.2)	42 (77. 8)	0.91	[0.43, 1.95]		0.64 (0.29, 1.42)
35–39	8 (16.7)	40 (83.3)	0.69	[0.29, 1.61]		0.56 (0.22, 1.44)
≥ 40	20 (35.1)	37 (64.9)	1.44	[0.74, 2.82]		1.04 (0.53, 2.03)
**Level of education**						
Primary	5 (29.4)	12 (70.6)	1			
Secondary	18 (21.7)	65 (78.3)	0.74	[0.32, 1.72]		
Tertiary	24 (25.5)	70 (74.5)	0.87	[0.38, 1.96]		
Experience in mining sector						
1–5 years	29 (20.4)	113 (79.6)	1			1
> 5 years	20 (37.0)	34 (63.0)	1.81	[1.13, 2.92] *		1.52 (0.85, 2.74)
**Job demands**						
High	17 (73.9)	6 (26.1)	1		1	
Low	32 (18.5)	141 (81.5)	0.25	[0.17, 0.37] ***	0.18	[0.08, 0.44] *
**Job insecurity**						
High	20 (30.8)	45 (69.2)	1			
Low	29 (22.1)	102 (77.9)	0.72	[0.44, 1.17]		
**Job control**						
Yes	12 (38.7)	19 (61.3)	1			
No	37 (22.4)	128 (77.6)	0.58	[0.34, 0.98]		
**Work relationship**						
Good	15 (27.3)	40 (72.7)	1			
Bad	34 (24.1)	107 (75.9)	0.88	[0.52, 1.49]		
**Mental state**						
Not normal	7 (38.9)	11 (61.1)	1			1
Normal	42 (23.6)	136 (76.4)	0.61	[0.32, 1.15]		0.61 (0.30, 1.23)

## Discussion

With regards to the prevalence of MSDs, about 61.2% of workers had experienced MSD symptoms in at least one body region and 25.0% were work-related (WRMSDs). The prevalence of WRMSDs obtained in this study was similar to what has been previously reported among workers in the industrial sector which includes mining in Africa [12]. However, the reported prevalence of WRMSDs was lower compared to a similar study amongst workers in gold mines in Ghana (85.5%) [24] and among quarry workers in Southeast Nigeria (83.3%) [25]. The discrepancy could be explained by the possibility that Twangiza mining site might be better designed ergonomically. We also believe this could be an issue of under reporting by workers who may consider some complaints as non-significant. Since we involved supervisors in questionnaire distribution, it is also a possibility that some workers may have underreported MSD complaints for fear of reprisal or due to the thought that the survey could identify those with MSDs as not fit for work.

The body parts susceptible to WRMSDs in our study did not differ from those reported in other studies which reported lower back, shoulders and hands as body regions commonly involved in WRMSDs [26]. The lower back was the most affected body part in this study and it is supported by previous studies which highlight low-back pain as the most frequent WRMSD complaint in any industry [1, 2, 12, 24, 27–29]. Hip complaints were the second most recorded parts contrary to the other studies which reported the wrists/hands [2, 24]. Nevertheless, working in restricted postures such as performing a lift while lying on the ground may incur additional burdens on muscles of the hips and thighs hence higher complaints. Additionally, these workers may have limited knowledge about the work activity hence no effective mitigation measures, an area of research which needs further investigation in future studies.

A significant association was observed between working for > 9 h and reporting a WRMSD. This confirms earlier studies in different settings that indicated an association between long working time and complaints of WRMSDs [30, 31]. Increase in working hours per day also means increased exposure time to the physical demands during work. Longer work hours are indicators of high workload and have been identified to increase the risk of lower back pain and other WRMSDs [32]. Based on this, we suggest the need to design appropriate system level approaches to reduce exposure/work time for mining workers especially those in most demanding operations.

Heavy lifting and/or lowering/shovelling for over 2 h/day was significantly associated with reporting a WRMSD, which is consistent with other studies [24, 26, 33]. This is evident in low income countries where manual labour is used in physically demanding tasks and in most cases manual handling of heavy loads is almost inherent in the mining industry [34]. Heavy lifting and shovelling for prolonged periods involve risk factors such as highly repetitive motions, forceful exertions, vibration exposures, poor/awkward posture all of which have been shown to cause WRMSDs [14, 35]. This finding justifies the need for ergonomic training and education about prevention and mitigation of WRMSDs in the mining setting in DR Congo. Such trainings should inform the preventive actions specific for ergonomic risks of the different body parts.

In our study, the psychological factor (job demands) was associated with WRMSDs. Workers with low job demands were less likely to report WRMSDs. The effect of psychosocial factors has been implicated by researchers in the causation of WRMSDs [36]. Studies have suggested an association between high job demands and higher presentation of WRMSD symptoms [37, 38]. Higher demands might cause tense scheduling, panic and consequently expose workers to high risk of WRMSDs. Therefore, there is a need to optimize job design and ensure better physical and psychosocial demands of work. This can both improve productivity while mitigating incidences of WRMSDs.

The results showed no significant association between age and reporting a WRMSD which is in agreement with previous studies [39, 40]. Some studies have highlighted that reporting MSDs increases with age [41, 42]. With ageing, people become less able to put stress on muscles without risking injuries and are more susceptible to bones breaking [43]. The small sample size may have limited our power to detect this association. Non significant association between work experience and self-reported WRMSDs has also been reported in other similar studies [24, 44] but contrary to the findings by Egwuonwu et al. [25] who found significantly higher WRMSDs among longer serving workers than those with less years. Whereas this association couldn’t be verified, long exposures are known to increase the risk of some disorders of the neck and upper limbs and musculoskeletal disorders in general [45].

### Study limitations

As this study was cross sectional, we can not make causal inferences of the associated factors and WRMSDs. In addition, musculoskeletal symptoms were self-reported, and thus respondents can give vague responses or exaggerate their MSD complaints. The small sample size may also have limited the strength and significance of some associations. More rigorous designs such as prospective cohorts with sufficiently larger sample sizes may be required to provide more sound research evidence. Additionally, in our study, we did not exclude participants who previously underwent surgery, and overweight participants and these could inflate prevalence of WRMSDs. Nevertheless, the study adds to the growing body of evidence of the factors associated with WRMSDs in various work settings.

## Conclusions

The study found that WRMSDs are prevalent amongst workers of Twangiza gold mine. The body part most affected by WRMSDs was the lower back. The predictors of WRMSDs included hazardous exposure to lifting/lowering/shovelling and longer work shifts exceeding 9 h, while low demanding jobs were protective against WRMSDs. To prevent WRMSDs, there is need to train workers on ergonomics, reduce workload (hours per shift) and ensure optimal job design enabling better physical and psychosocial demands at work.

## Supplementary Information

**Additional file 1:.** Questionnaire (English Version)

## Data Availability

The data that support the findings of this study are available from the corresponding author, upon reasonable request.

## Abbreviations

DRC: Democratic Republic of Congo
MSD: Musculoskeletal disorder
PR: Prevalence ratios
WRMSD: Work related Musculoskeletal disorder
