# Supplementary material for: Prevalence and predictors of work-related musculoskeletal disorders among workers of a gold mine in south Kivu, Democratic Republic of Congo
Source: BMC Musculoskelet Disord. 2020 Dec 1;21:797. doi: 10.1186/s12891-020-03828-8 (PMC7709424; doi:10.1186/s12891-020-03828-8)
Supplement: Supplementary file 1 — Additional file 1:. Questionnaire (English Version) [file 12891_2020_3828_MOESM1_ESM.docx]

# QUESTIONNAIRE (ENGLISH VERSION)

**Responding to the questionnaire**

Date ……………

On the following pages are questions about your work and the organization where you work. The purpose of this questionnaire is to collect the information needed to identify risk factors for musculoskeletal disorders at your work place. It is also hoped that the information collected will help develop your work and the work environment. Please take your time answering. Answer all by choosing the alternative that best describes your opinion. Be assured that the information you give shall be kept confidential and anonymous. Mark the choice that best applies to your situation with a ✓ *or* **X**

**SECTION A**

**DEMOGRAPHIC DATA**

**1**. Sex: Male🞎 1 Female 🞎2 **2.** Weight………..Kg **3.**Height……..M **4**. Age…………Years

**5.** Married: NO 🞎 1 YES 🞎 2

**6**. Highest level of education: No school🞎 Primary🞎 Secondary🞎 College🞎 University🞎

**7**. Income per month: Less than 500 USD 🞎 1 More than 500 USD 🞎 2

**8**. Title of occupation: ……..…………...………………………………………………………….

**9**. How long have you worked for this mine…………………..Years: Since: ….… …….

**10**. In what department/Section/Unit do you work? ……………………………………………….

Department…………………………………… Section: ………….…………………….

**11**. How many years have you been doing your present kind of work? …..Years Since: …….

**12**. Is your employment contract permanent Yes 🞎 1 No 🞎 2

**13**. Is your job a supervisory position? Yes 🞎 1 No 🞎 2

**14**. How many hours per shift or day do you work? ……………………………………

Section B: 1: This part of the questionnaire is designed to determine the musculoskeletal (muscle bone) symptoms among workers. Note that the questionnaire is to be answered even if you have never had trouble (ache, pain, numbness, injury) in any part of your body.

| **To be answered by everyone** | | | **To be answered by those who have had trouble** |
| --- | --- | --- | --- |
| **1**. Have you at any time during the last 12months had trouble (ache, pain, numbness, injury) in any of the following parts of the body?  🞎 1. No 🞎 2. Yes  **If No; proceed to section C (Next page)**  **If YES; circle the part or parts affected and proceed to question 2** | | | **2.** Did the trouble (ache, pain, numbness, injury) start while at work?  🞎 1. No 🞎 2. Yes |
|  |  |  | **3**. Did you report the trouble as arising from work?    🞎 1. No 🞎 2. Yes |
| a. Neck |  | 1. No |  |
|  |  | 2. Yes |  |
| b. Shoulder | | 1. No  2. Yes, right shoulder  3. Yes, left shoulder  4. Yes, both shoulders | **4**. Were you treated at the site clinic for the above mentioned problem?  🞎 1. No 🞎 2. Yes |
|  |  |  | **5**. Have you at any time during the last 12months been prevented from doing your normal work (at home or away from home) because of the trouble?  🞎 1. No 🞎 2. Yes |
| c. Elbows | | 1. No  2. Yes, right elbow  3. Yes, left elbow  4. Yes, both elbows |  |
| d. Wrists/Hands | | 1. No  2.Yes,right wrist/hand  3.Yes,left wrist/hand  4.Yes,both wrists/hand |  |
|  |  |  | **6**. What do you think caused the pain/injury/ ache/numbness?............................................................................................................................................................................................................................................................................................................................................................................................................................................................................................................................................................................................................................................................................................................................................. |
| e. Upper back | | 1. No  2. Yes |  |
| f. Lower back | | 1. No  2. Yes |  |
| g. One or both hips/thighs | | 1. No  2. Yes |  |
| h. One or both knees | | 1. No  2. Yes |  |
| i. One or both ankles/feet | | 1. No  2. Yes |  |

Section C: 1 This part of the questionnaire will measure the physical aspects of your work. Circle or tick the choice that best applies to your job.

| **1.Heavy or Frequent Lifting / Lowering / Shoveling** | | | | | | |
| --- | --- | --- | --- | --- | --- | --- |
| 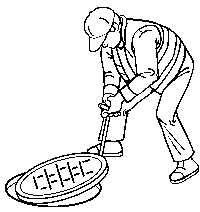  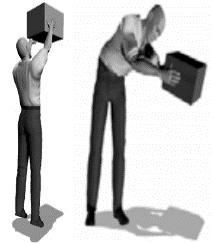 | 1. **a.** Do you lift ***or*** lower objects weighing more than 50Kgs? | | | | | |
|  |  | Never |  | Occasionally |  |  |
|  |  | Less than10 times per day |  | More than 10times per day |  |  |
|  | **b**. Do you lift ***or*** lower objects weighing more than 25kgs? | | | | | |
|  |  | Never |  | Occasionally |  |  |
|  |  | Less than10 times per day |  | More than 10times per day |  |  |
|  |  | | | | | |
| 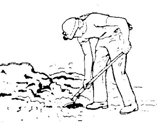 | \| Never \|  \| Occasionally \|  \|  \|  \| \| --- \| --- \| --- \| --- \| --- \| --- \| \| Less than 2 hours per day \|  \| 2 - 4hrs per day \|  \| More than 4hrs per day \|  \|   **c**. Do you do a lot of Shoveling**?** | | | | | |

| **2. Awkward Postures** | | | |
| --- | --- | --- | --- |
|  | | \| Never \|  \| Occasionally \|  \|  \|  \| \| --- \| --- \| --- \| --- \| --- \| --- \| \| Less than 2 hours per day \|  \| 2 - 4hrs per day \|  \| More than 4hrs per day \|  \|   **a**. Do you work with hand(s) above the head?  Total/day | |
| 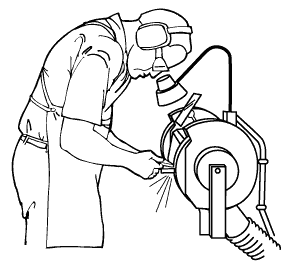 | | \| Never \|  \| Occasionally \|  \|  \|  \| \| --- \| --- \| --- \| --- \| --- \| --- \| \| Less than 2 hours per day \|  \| 2 - 4hrs per day \|  \| More than 4hrs per day \|  \|   **b**. Do you Work with the neck bent more than 30 degrees (without support)?  Total/day | |
| 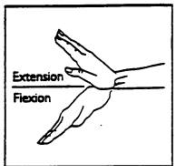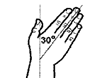 | | **c**. Do you work with a bent wrist(s)?   \| Never \|  \| Occasionally \|  \|  \|  \| \| --- \| --- \| --- \| --- \| --- \| --- \| \| Less than 2 hours per day \|  \| 2 - 4hrs per day \|  \| More than 4hrs per day \|  \|   Total/day | |
| 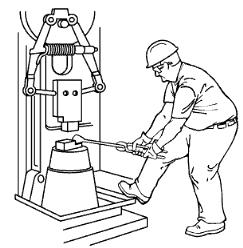 | | | **d.** Do you work with the back bent (without support)?   \| Never \|  \| Occasionally \|  \|  \|  \| \| --- \| --- \| --- \| --- \| --- \| --- \| \| Less than 2 hours per day \|  \| 2 - 4hrs per day \|  \| More than 4hrs per day \|  \|   Total/day |
| 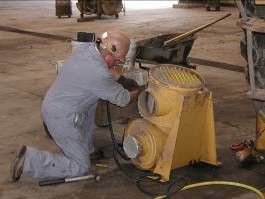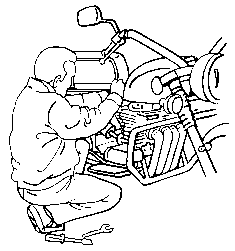 | | | **a**. Does your job involve Squatting?   \| Never \|  \| Occasionally \|  \|  \|  \| \| --- \| --- \| --- \| --- \| --- \| --- \| \| Less than 2 hours per day \|  \| 2 - 4hrs per day \|  \| More than 4hrs per day \|  \|   Total/day  **b**. Does your job involve Kneeling?   \| Never \|  \| Occasionally \|  \|  \|  \| \| --- \| --- \| --- \| --- \| --- \| --- \| \| Less than 2 hours per day \|  \| 2 - 4hrs per day \|  \| More than 4hrs per day \|  \|   Total/day |
| **3. High Hand Force - Pinch and power Grip** | | | |
| 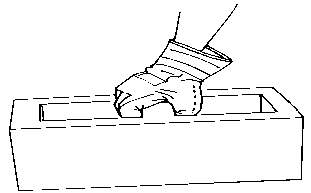   | | | \| Never \|  \| Occasionally \|  \|  \|  \| \| --- \| --- \| --- \| --- \| --- \| --- \| \| Less than 2 hours per day \|  \| 2 - 4hrs per day \|  \| More than 4hrs per day \|  \|   **a.** Do you Pinch unsupported objects?  Total/day  **b.** what objects do you pick up with a pinch grip? …………………………………………………….. |
|    | | | **b.** Do you grasp an unsupported object(s) weighing 5 or more Kg per hand, ***or*** grasping with a forceful grip?   \| Never \|  \| Occasionally \|  \|  \|  \| \| --- \| --- \| --- \| --- \| --- \| --- \| \| Less than 2 hours per day \|  \| 2 - 4hrs per day \|  \| More than 4hrs per day \|  \|   Total/day |
| 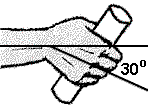 | | | **c.** Does your job involve grasping objects with wrists bent?   \| Never \|  \| Occasionally \|  \|  \|  \| \| --- \| --- \| --- \| --- \| --- \| --- \| \| Less than 2 hours per day \|  \| 2 - 4hrs per day \|  \| More than 4hrs per day \|  \|   Total/day |
| **4. Highly Repetitive Work** | | | |
|    | **a**. Does your work involve repeating the same motion with little or no variation every few seconds?   \| Never \|  \| Occasionally \|  \|  \|  \| \| --- \| --- \| --- \| --- \| --- \| --- \| \| Less than 2 hours per day \|  \| 2 - 4hrs per day \|  \| More than 4hrs per day \|  \|     Total/day | | |
| **5. Vibrating Tools (Hand-Arm Vibration)** | | | |
| 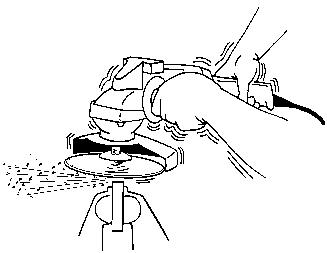 | \| Never \|  \| Occasionally \|  \|  \|  \| \| --- \| --- \| --- \| --- \| --- \| --- \| \| Less than 2 hours per day \|  \| 2 - 4hrs per day \|  \| More than 4hrs per day \|  \|   **a**. Does your work involve using grinders, jig saws or other hand tools that typically have moderate vibration levels?  Total/day | | |
|  | **b.** Does your work involve using impact wrenches, chain saws, percussive tools (jack hammers, scalers, chipping hammers) or other tools that typically have high vibration levels?   \| Never \|  \| Occasionally \|  \|  \|  \| \| --- \| --- \| --- \| --- \| --- \| --- \| \| Less than 30 minutes/day \|  \| More than 30 minutes \|  \|  \|  \|   Total/day | | |
| **6. Bouncing or Jarring (Whole Body Vibration)** | | | |
|  | a. Does your work involve operating mobile equipment?   \| Never \|  \| Occasionally \|  \|  \|  \| \| --- \| --- \| --- \| --- \| --- \| --- \| \| Less than 2 hours per day \|  \| 2 - 4hrs per day \|  \| More than 4hrs per day \|  \|   b. Name Equipment ________________________________  c. I travel over rough roads (circle one)  **1**🞎. Never **2**🞎. Sometimes **3**🞎. Most of the time.  **4**🞎. All the time. | | |
| \| **7. Static postures** \| \| --- \| | | | |
|  | **a.** Does your work involve Standing without changing position?   \| Never \|  \| Occasionally \|  \|  \|  \| \| --- \| --- \| --- \| --- \| --- \| --- \| \| Less than 2 hours per day \|  \| 2 - 4hrs per day \|  \| More than 4hrs per day \|  \|  \| Never \|  \| Occasionally \|  \|  \|  \| \| --- \| --- \| --- \| --- \| --- \| --- \| \| Less than 2 hours per day \|  \| 2 - 4hrs per day \|  \| More than 4hrs per day \|  \|   **b.** Does your work involve Sitting without changing position? | | |
|  |  |  |  |
| **8. Pushing and Pulling** | | | |
|  | \| Never \|  \| Occasionally \|  \|  \|  \| \| --- \| --- \| --- \| --- \| --- \| --- \| \| Less than 8 times/day \|  \| 8 – 30 times per day \|  \| More than 30 times per day \|  \|   Does your work involve pushing against an object, such as a trolley  with a ***maximum effort*** (body leaning with bent legs into the push  Does your work involve pushing against objects, like a trolley with a ***moderate effort*** (body slightly leaning with straight legs into the push).   \| Never \|  \| Occasionally \|  \|  \|  \| \| --- \| --- \| --- \| --- \| --- \| --- \| \| Less than 16 times \|  \| 16 – 50 times \|  \| More than 50 times \|  \| | | |
|    | Does your work involve pulling against objects, like a/an electrical cable, fuel hose or trolley with a ***maximum effort*** (body leaning with bent legs into the pull)?   \| Never \|  \| Occasionally \|  \|  \|  \| \| --- \| --- \| --- \| --- \| --- \| --- \| \| Less than 8 times \|  \| 8 – 30 times \|  \| More than 30 times \|  \|   Does your work involve pulling against an object, such as a/an electrical cable, fuel hose, or trolley with a ***moderate effort*** (body slightly leaning with straight legs into the pull).   \| Never \|  \| Occasionally \|  \|  \|  \| \| --- \| --- \| --- \| --- \| --- \| --- \| \| Less than 16 times \|  \| 16 – 50 times \|  \| More than 50 times \|  \| | | |

**JOB DEMANDS**

SECTION D. 1 This part of the questionnaire will measure the psychosocial aspect of your work. Please choose only one answer for each statement. Circle the choice that best describes your opinion as shown below

| **1.** **How much do you agree or disagree with these statements?** | **Strongly disagree** | **Disagree** | **Agree** | **Strongly agree** |
| --- | --- | --- | --- | --- |
| a. My job requires working very fast | 1 | 2 | 3 | 4 |
| b. My job requires working very hard | 1 | 2 | 3 | 4 |
| c. I am NOT asked to do an excessive amount of  work | 1 | 2 | 3 | 4 |
| d. I have enough time to get the job done | 1 | 2 | 3 | 4 |
| e. My job requires that I learn new things | 1 | 2 | 3 | 4 |
| f. I am able to influence the availability of equipment needed to do my work. | 1 | 2 | 3 | 4 |
| g. I can take a break when I want to | 1 | 2 | 3 | 4 |

SECTION D. 2 Job Satisfaction and Security

| **2.** **How much do you agree or disagree with these statements?** | **Strongly disagree** | **Disagree** | **Agree** | **Strongly**  **agree** |
| --- | --- | --- | --- | --- |
| a. My supervisor is willing to listen to my work related problems. | 1 | 2 | 3 | 4 |
| b. I have job security | 1 | 2 | 3 | 4 |
| c. My job requires a great deal of concentration | 1 | 2 | 3 | 4 |
| d. In my job, there is constant pressure from my  work group to keep up | 1 | 2 | 3 | 4 |
| e. My employer cares about my health and safety on  the job | 1 | 2 | 3 | 4 |
| f. I receive the training I need to do my job well. | 1 | 2 | 3 | 4 |

3. How little or how much influence or control do you have over aspects of your work?

SECTION D. 3 Job Control

| **How much influence do you have over** | **Very little** | **Little** | **Moderate amounts** | **Much** | **Very Much** |
| --- | --- | --- | --- | --- | --- |
| a. The variety of tasks you perform? | 1 | 2 | 3 | 4 | 5 |
| b. The amount of work you do? | 1 | 2 | 3 | 4 | 5 |
| c. The pace of your work, that is how fast or slow you work? | 1 | 2 | 3 | 4 | 5 |
| d. The hours that you work? | 1 | 2 | 3 | 4 | 5 |

SECTION D. 4 Work Relationship

| **4. How much support do you receive on your job?** | **Very much or (very easy)** | **Much or (easy)** | **A little** | **Not at all** |
| --- | --- | --- | --- | --- |
| a. How much can your immediate supervisor be relied upon when things get tough at work? | 1 | 2 | 3 | 4 |
| b. How easy is it to talk with your immediate supervisor (boss)? | 1 | 2 | 3 | 4 |
| c. How much can your co-workers be relied upon when things get tough at work? | 1 | 2 | 3 | 4 |
| d. How easy is it to talk with your co-workers | 1 | 2 | 3 | 4 |

**5. How have you felt in the past month including today?**

SECTION D. 5 Mental State (self-evaluation)

| During the past month: | **Rarely or none of the time** | **Sometimes** | **Often** | **Most or all of the time** |  |
| --- | --- | --- | --- | --- | --- |
| a. I felt that everything I did was an effort | 1 | 2 | 3 | 4 |  |
| b. I was happy. | 1 | 2 | 3 | 4 |  |
| c. I felt depressed. | 1 | 2 | 3 | 4 |  |
| d. People were unfriendly. | | 1 | 2 | 3 | 4 |
| e. I felt nervous. | | 1 | 2 | 3 | 4 |
